# Expression of Concern: The effects of learning experience on college students’ deep english learning: A study of the chain mediation effect of motivation and strategy

**DOI:** 10.1371/journal.pone.0354764

**Published:** 2026-07-28

**Authors:** The PLOS One Editors

After this article [1] was published, the *PLOS One* Editors identified concerns about authorship.

In addition, the articles cited as References 18 and 20 were retracted after [1] was published.

In light of these issues, the *PLOS One* Editors issue this Expression of Concern. Readers are advised to interpret the article [1] with caution.
