# Supplementary material for: The effects of learning experience on college students’ deep english learning: a study of the chain mediation effect of motivation and strategy
Source: PLoS One. 2025 Jun 4;20(6):e0325491. doi: 10.1371/journal.pone.0325491 (PMC12136437; doi:10.1371/journal.pone.0325491)
Supplement: Attachment — Submitted filename: Response_to_Reviewers.pdf [file pone.0325491.s003.pdf]

Response to Reviewers - Manuscript PONE-D-25-12036

Dear Dr. Butt and Reviewers,

We sincerely appreciate the valuable feedback and suggestions provided by the reviewers and the editor. The comments have been instrumental in helping us improve the quality and clarity of our manuscript. Below, we provide a detailed point-by-point response to the reviewers' comments and concerns.

Response to Reviewer #1

Comment 1: Reviewer #1 mentioned that the study is technically sound and the data supports the conclusions.

Response: We are grateful for Reviewer #1's positive assessment of our study's technical soundness and the adequacy of the data in supporting our conclusions. This affirmation has given us confidence in the overall direction of our research.

Response to Reviewer #2

Comment 1: Reviewer #2 suggested that the abstract should be rewritten to exclude redundant text and focus solely on objectives, methods, data, results, and conclusions.

Response: We have thoroughly revised the abstract to remove redundant text and ensure it succinctly captures the study's objectives, methods, data, results, and conclusions. The revised abstract now adheres to the PLOS ONE format and provides a clearer overview of the study.

Comment 2: Reviewer #2 raised questions about the selection of samples based on gender, age, educational background, and academic achievement levels and sought clarification on whether the analysis was done on an overall basis or sample-based.

Response: We have added detailed explanations regarding the selection of samples and the rationale behind including participants with different genders, ages, educational backgrounds, and academic achievement levels. We have also clarified that the analysis was conducted on an overall basis, with comparisons made where appropriate. These clarifications are provided in the revised manuscript.

Comment 3: Reviewer #2 requested further details on the educational background of the participants, specifically whether it refers to the educational background of the parents/family members or the students themselves.

Response: We have specified in the manuscript that the educational background refers to the students' own educational qualifications, including their degree programs and fields of study.

Comment 4: Reviewer #2 suggested that the research gap should be provided in the beginning of the abstract.

Response: We have revised the abstract to include a brief statement on the research gap, highlighting the limited existing research on the chain mediation effect of motivation and strategy in the context of learning experience and deep English learning among college students.

Comment 5: Reviewer #2 pointed out that the introduction should provide a more detailed literature review and clarify the significance of the research question.

Response: We have expanded the literature review in the introduction to include more relevant studies and to better establish the significance of the research question. We have also specified whether the research gap is global or specific to a particular region.

Comment 6: Reviewer #2 requested further clarification on the research sample, including the meaning of "comprehensive universities," the rationale for selecting the sample, and the method of determining the sample size.

Response: We have provided additional details about the meaning of "comprehensive universities" and the rationale for selecting the sample. We have also explained the method used to determine the sample size and the number of universities involved in the study.

Comment 7: Reviewer #2 asked whether the questionnaire used in the study was adopted from the literature or newly designed, and if newly designed, whether its reliability and validity were tested.

Response: We have clarified that the questionnaire was newly designed and that its reliability and validity were tested through pretesting and various statistical analyses. The results of these tests are now included in the manuscript.

Comment 8: Reviewer #2 found Table 1 confusing and requested reformatting and restructuring for clarity.

Response: We have thoroughly restructured and reformatted Table 1 to enhance clarity. We have also provided more descriptive labels for the categories to improve comprehension.

Comment 9: Reviewer #2 requested more information on the statistical methods used, including the specific purposes of using SPSS and AMOS software.

Response: We have added detailed explanations of the statistical methods used, specifying the roles of SPSS and AMOS software in the data analysis process.

Comment 10: Reviewer #2 suggested that the discussion section should compare the study's findings with previous studies rather than merely reproducing results.

Response: We have revised the discussion section to include more comparisons with previous studies and to better highlight the contributions of our findings to the existing literature.

Comment 11: Reviewer #2 noted that the conclusion section was too lengthy and repetitive.

Response: We have streamlined the conclusion section to focus on the key findings and implications of the study, removing redundant information and ensuring it concisely addresses the study's contributions.

Sincerely,

Zhang Qun
Jin Hao
Teng Wenwen
