# Supplementary material for: The effects of learning experience on college students’ deep english learning: a study of the chain mediation effect of motivation and strategy
Source: PLoS One. 2025 Jun 4;20(6):e0325491. doi: 10.1371/journal.pone.0325491 (PMC12136437; doi:10.1371/journal.pone.0325491)
Supplement: Attachment — Submitted filename: PLOSOne_Human_Subjects_Research_Checklist.docx [file pone.0325491.s002.docx]

**Human Participants Research Checklist**

***Complete the following if your study involved human participants or human participants’ data. These questions should be addressed for prospective and retrospective studies.***

1. Did you obtain ethics approval for this study?
   - If yes, please upload (file type “Other”) the original approval document you received from your ethics committee. If the original document is in another language, please also provide an English translation.

___ Uploaded	_**√**_ N/A

- If you did not obtain ethical approval, please explain why this was not required below.

This study collected data through a questionnaire survey and did not involve any interventions or operations that may have a negative impact on the physical or mental health of participants. The data collected in the study is anonymous, does not contain any personally identifiable information, and does not involve sensitive personal information or commercial interests. The research aims to understand the relationship between learning experiences, motivations, and strategies and deep learning outcomes, without involving harm to individuals, sensitive information, or business interests. According to Chapter 3, Article 32 of the Measures for Ethical Review of Life Science and Medical Research Involving Human Subjects, this study meets the conditions for exemption from ethical review.
**https://www.gov.cn/zhengce/zhengceku/2023-02/28/content_5743658.htm**

2. If you prospectively recruited human participants for the study – for example, you conducted a clinical trial, distributed questionnaires, or obtained tissues, data or samples for the purposes of this study, please report in the Methods:
   i. the day, month and year of the **start and end** of the recruitment period for this study.
   ii. whether participants provided informed consent, and if so, what type was obtained (for instance, written or verbal, and if verbal, how it was documented and witnessed). If your study included minors, state whether you obtained consent from parents or guardians. If the need for consent was waived by the ethics committee, please include this information.

__**√**_ Completed	___ N/A

Date of distribution of questionnaire: the distribution of questionnaire for this study was from 1st October 2024 to 31st January 2025.Informed consent: the researcher clearly stated the purpose and content of the study on the questionnaire homepage. All participants were adults and no minors were involved. Prior to the survey, the researcher verbally consulted the person completing the questionnaire for consent and the respondents agreed to participate and understand the content of the questionnaire

3. If you are reporting a retrospective study of medical records or archived samples, please report in the Methods section:
i. the day, month and year when the data were accessed for research purposes
ii. whether authors had access to information that could identify individual participants during or after data collection

___ Completed	_**√**_ N/A

This study did not involve a retrospective study of medical records or archived samples
